# Promising antifungal activity of new oxadiazole against *Candida krusei*

**DOI:** 10.1371/journal.pone.0227876

**Published:** 2020-01-14

**Authors:** Daniella Renata Faria, Karina Mayumi Sakita, Isis Regina Grenier Capoci, Glaucia Sayuri Arita, Franciele Abigail Vilugron Rodrigues-Vendramini, Admilton Gonçalves de Oliveira Junior, Maria Sueli Soares Felipe, Patrícia de Souza Bonfim de Mendonça, Terezinha Inez Estivalet Svidzinski, Erika Seki Kioshima

**Affiliations:** 1 Department of Clinical Analysis and Biomedicine, Laboratory of Medical Mycology, State University of Maringá, Maringá, Paraná, Brazil; 2 Department of Microbiology, Laboratory of Microbial Biotechnology, State University of Londrina, Londrina, Paraná, Brazil; 3 Department of Cell Biology, Laboratory of Molecular Biology, University of Brasília, Brasília, Distrito Federal, Brazil; Louisiana State University, UNITED STATES

## Abstract

*Candida krusei* is one of the most common agents of invasive candidiasis and candidemia worldwide, leading to high morbidity and mortality rates. This species has become a problem due to its intrinsic resistance and reduced susceptibility to azoles and polyenes. Moreover, the number of antifungal drugs available for candidiasis treatment is limited, demonstrating the urgent need for the discovery of novel alternative therapies. In this work, the *in vivo* and *in vitro* activities of a new oxadiazole (LMM11) were evaluated against *C*. *krusei*. The minimum inhibitory concentration ranged from 32 to 64 μg/mL with a significant reduction in the colony forming unit (CFU) count (~3 log_10_). LMM11 showed fungicidal effect, similar to amphotericin, reducing the viable cell number (>99.9%) in the time-kill curve. Yeast cells presented morphological alterations and inactive metabolism when treated with LMM11. This compound was also effective in decreasing *C*. *krusei* replication inside and outside macrophages. A synergistic effect between fluconazole and LMM11 was observed. *In vivo* treatment with the new oxadiazole led to a significant reduction in CFU (0.85 log_10_). Furthermore, histopathological analysis of the treated group exhibited a reduction in the inflammatory area. Taken together, these results indicate that LMM11 is a promising candidate for the development of a new antifungal agent for the treatment of infections caused by resistant *Candida* species such as *C*. *krusei*.

## Introduction

Fungal infections have emerged worldwide, accounting for about 6% of all healthcare-associated infections (HAIs), especially in the increasing population of immunocompromised patients [1–5]. Invasive candidiasis and candidemia represent a serious public health problem associated with high mortality rates, ranging from 40 to 85%, prolonged length of hospital stay and high costs [6–9]. For many years, *Candida albicans* was the most frequent agent isolated from clinical specimens. However, the epidemiology has changed with an increase in infection rates with *Candida* non-*C*. *albicans* (CNCA) species [8, 10–12]. Studies have shown that approximately 55 to 65% of candidemia cases are caused by CNCA species [8, 13, 14].

*Candida krusei* has become an emerging pathogen, responsible for about 1.5 to 8% of candidemia cases. This is worrisome due to its intrinsic resistance to fluconazole (FLC) and reduced susceptibility to other azoles and polyenes [8, 15–17]. Increased *C*. *krusei* infection rates have been associated with the prophylactic use of FLC and have facilitated the selection of pathogenic fungi resistant to these agents [18]. Echinocandins are a good therapeutic option for the treatment of invasive *C*. *krusei* infection. However, studies have shown the rapid acquisition of resistance during treatment with caspofungin [19–21]. The toxicity and the variable effectiveness of the antifungal drugs available for candidiasis treatment demonstrate the urgent need for the discovery of novel antifungal agents [21–24].

*In silico* approaches have explored virtual screening of chemical libraries against pathogen-specific targets for drug discovery. This strategy has contributed to reducing the time and costs associated with drug development [24]. Thioredoxin reductase (Trr1) is a promising target, which acts primarily in resistance to oxidative stress [25]. Several potential Trr1 ligands have been selected and tested against important pathogenic fungi such as *Candida* spp., *Cryptococcus neoformans* and *Paracoccidioides* spp. [26–29]. Two hit compounds presented selective antifungal activity, including the compound LMM11, which belongs to the oxadiazole class [27–29]. Therefore, in this study, the *in vitro* and *in vivo* antifungal activity of LMM11 was evaluated against *C*. *krusei*.

## Materials and methods

### Organisms

Eighteen clinical isolates of *C*. *krusei* from hospitalized patients (12 urine, four blood, one catheter tip and one bronchoalveolar lavage) and the reference strain *C*. *krusei* ATCC 6258 (American Type Culture Collection) were used. They belong to the archive collection of the Medical Mycology Laboratory of the State University of Maringá, Paraná, Brazil (Human Research Ethics Committee COPEP no. 2.748.843). Except for the minimal inhibitory concentration determination and the checkerboard assay, all experiments were performed with the reference strain of *C*. *krusei* only.

In each experiment, the yeast was subcultured on Sabouraud dextrose agar (SDA, Difco^tm^, Detroit, MI, USA) at 35°C for 24 hours. The cellular density was adjusted using a Neubauer chamber before each assay.

### Compound

The compound LMM11 (4-[cyclohexyl(ethyl)sulfamoyl]-N-[5-(furan-2-yl)- 1,3,4-oxadiazol-2-yl]benzamide), which belongs to the oxadiazole class, was commercially purchased from Life Chemicals Inc. (Burlington, ON, Canada) [28]. The stock solutions were prepared in dimethyl sulfoxide (DMSO) at a concentration of 100 μg/mL. Pluronic® F-127 was used to increase compound solubility.

### Minimum inhibitory and fungicidal concentration assays

The antifungal activity of LMM11 was evaluated by determining the minimal inhibitory concentration (MIC), based on the broth microdilution method, according to Clinical Laboratory Standards Institute (CLSI) document M-27A3 [30], with modifications. Briefly, LMM11 was diluted in RPMI-1640 medium (Gibco/Invitrogen, Grand Island, NY, USA) at concentrations ranging from 0.5 to 256 μg/mL. The initial inoculum (2–3 ×10^6^) was adjusted in saline using a Neubauer chamber. Subsequently, a 1:50 dilution in saline and 1:20 dilution in RPMI-1640 were performed. The inoculum of 2–3 × 10^3^ yeast cells/mL was diluted 1:2 into 96-well plates containing different concentrations of LMM11. The negative control was medium only without inoculum and the positive control was medium plus inoculum. The incubation time was 24 hours at 35°C. The MIC values were determined by measuring the absorbance at 405 nm on a microplate reader (Expert Plus, ASYS, UK) and defined as the lowest LMM11 concentration able to inhibit growth equal to or higher than 50% in relation to the positive control. The MIC was also determined for voriconazole (VRC; 0.032–16 μg/mL; Pfizer, Brazil) and fluconazole (FLC; 0.125–64 μg/mL; Pfizer, Brazil) according to M27-A3. The lowest concentration of the antifungal agent that was able to inhibit growth by 50% relative to the positive control was considered the MIC. The cut-off levels for susceptible (S), dose-dependent susceptible (DDS) and resistant (R) were determined in accordance with the M27-S4 document [31].

The minimal fungicidal concentration (MFC) was evaluated after yeast exposure to LMM11 (0.5 to 256 μg/mL) as described above. Aliquots (3 μL) from each well from the MIC microplates were transferred to SDA plates and incubated at 35°C for 24 hours. The MFC was defined as the lowest LMM11 concentration at which ≤ 1 colony was visible on the agar plate. In addition, for each LMM11 concentration tested against *C*. *krusei* ATCC 6258, the number of colony forming units per milliliter (CFU/mL) was quantified. Aliquots (200 μL) from each well of the MIC microplates were diluted (10^1^, 10^2^, 10^3^, 10^4^ and 10^5^) in phosphate buffered saline (PBS), then 20 μL of each dilution was plated on SDA and incubated at 35°C for 24 h prior to colony counting.

### Time-kill curve

The time-kill curve was determined as previously described by Klepser *et al*. [32], with some modifications. The inoculum of *C*. *krusei* ATCC 6258 was adjusted to 2–3× 10^3^ yeast/mL in RPMI-1640 medium and treated with three LMM11 concentrations, i.e. 16 μg/mL, 32 μg/mL and 64 μg/mL. Untreated yeast cells were used as the drug-free control. FLC and amphotericin B (AmB; Sigma-Aldrich, Brazil) were used as conventional drug controls (MIC 16 μg/mL and 0.25 μg/mL, respectively). The suspensions were incubated in 24-well plates at 35°C. Aliquots of 100 μL were withdrawn, at predetermined time points (0, 2, 4, 6, 8, 12, 24, 28 and 36 h), diluted in PBS (10^1^, 10^2^, 10^3^, 10^4^ and 10^5^) and 20 μL of each dilution were plated on SDA and incubated at 35°C for 24 h for CFU determination.

### Fluorescent staining for yeast viability

A commercial LIVE/DEAD yeast viability kit (L-7009; Molecular Probes, Leiden, The Netherlands) was used to analyze yeast metabolic activity after treatment with LMM11 (for 24 hours) at the concentrations 16 μg/mL, 32 μg/mL and 64 μg/mL. In the dead control, the yeast were treated with 70% alcohol for 15 minutes. Untreated yeast cells were used as live control. Yeast cells were suspended in MOPS buffer containing 2% glucose. FUN-1 (10 μM) and Calcofluor White M2R (12.5 μM) cell dyes were added to the yeast cell suspensions. After incubation in the dark at 30°C for 30 min, the stained yeast was analyzed with an inverted fluorescence microscope (EVOS FL Cell Imaging System, Life Technologies, CA, USA), using appropriate filter sets, at x 400 magnification. The viability of fungal cells was determined by fluorescence analysis in at least 20 fields. Staining and the interpretation of fluorescence were performed according to the manufacturer’s instructions. Metabolically active cells showed red fluoresce in their structures while dead cells or cells with little or no metabolic activity exhibited diffuse bright green cytoplasmic fluorescence with no discernable red structures [33].

### Scanning electron microscopy

Scanning electron microscopy (SEM) was performed based on the protocol described by Oliveira [34]. *C*. *krusei* ATCC 6258 (2–3×10^3^ cells/mL in RPMI-1640 medium) was exposed to LMM11 at the concentrations of 16 μg/mL, 32 μg/mL, 64 μg/mL and incubated in 24-well plates at 35°C for 24 h. The cells were harvested, washed twice with PBS and fixed by immersion in 2.5% glutaraldehyde and 2% paraformaldehyde in 0.1 M sodium cacodylate buffer. Drops of the poly-L-lysine solution were placed onto clean polystyrene coverslips and allowed to form a uniform layer, then were left in the greenhouse (50°C) to dry (2 hours). The samples were washed three times with cacodylate buffer and the pellet was resuspended in a final volume of 1 mL. The suspension was applied to the coverslips and allowed to adhere for 1 hour at room temperature. The samples were washed with cacodylate buffer, dehydrated in an ethanol series (70%, 80%, 90% and 100%) and coated with gold (Baltec SDC 050 sputter coater) for observation using a scanning electron microscope (FEI Quanta 200) at 6000x and 12000x magnification.

### Transmission electron microscopy

Transmission electron microscopy (TEM) was performed after the treatment of *C*. *krusei* ATCC 6258 (2–3×10^3^ cells/mL in RPMI-1640 medium) with 32 μg/mL LMM11 in 24-well plates for 24h at 35°C. Then, the samples were harvested, washed twice with PBS and fixed with 2.5% glutaraldehyde in 0.1 M sodium cacodylate buffer. The cells were post-fixed in a solution containing 1% OsO4 (osmium tetroxide), 0.8% potassium ferrocyanide and 10 mM CaCl_2_ in 0.1 M cacodylate buffer, dehydrated in an increasing acetone gradient and embedded in Spurr resin (low viscosity embedding medium in Spurr’s kit). Ultrathin sections were stained with uranyl acetate and lead citrate and images were obtained on a Zeiss 900 TEM.

### Antifungal activity in defense cell against *C*. *krusei*

J774-A1 macrophage cells pre-adhered to 24-well plates were incubated with yeast cells at a 1:1 ratio for 2 hours. After incubation, when 60 to 80% of macrophages had at least one *C*. *krusei* cell internalized, the wells were washed to remove non-phagocytosed yeasts and LMM11 (32 μg/mL) was added to each well (treated). RPMI-1640 medium was used as the control (untreated). The plates were incubated at 37°C with 5% CO_2_. After 24 h, extracellular yeast cells were collected by washing and plated onto SDA agar plates for counting. Macrophages were also lysed with sterile cold water and plated onto SDA plates for intracellular fungal cell counts [35].

### Checkerboard assay

The combined effect of FLC (0.25–256 μg/mL) with LMM11 (1–64 μg/mL) was evaluated against a reference strain and one clinical isolate (selected according to high MIC values for FLC and VRC). The LMM11 compound was distributed and diluted vertically while FLC was added horizontally [36]. A yeast suspension of 2–3×10^3^ cells/mL was added to 96-well plates and incubated at 35°C for 24 hours. Inhibition was determined by measuring the absorbance at 405 nm. The synergistic interaction between FLC and LMM11 was determined based on the value of the fractional inhibitory concentration (FIC) that was calculated as the sum of FIC^A^ + FIC^B^, where A is the conventional drug and B is the novel compound. FIC^A^ is calculated as the ratio between MIC^A^ combined/MIC^A^ alone, while FIC^B^ is MIC^B^ combined/MIC^B^ alone. FIC values < 0.5 indicate a strongly synergistic effect, FIC < 1 a synergistic effect, FIC = 1 an additive effect, 1 < FIC < 2 no effect and FIC > 2 an antagonistic effect [37].

### Ethical aspects

The procedures were carried out in accordance with the regulations of the Institutional Ethics Committee for animal experimentation of the State University of Maringá, Brazil (Approval No. CEUA 9810191015, 04/22/2016). The animals were treated according to the Guidelines for the Care and Use of Laboratory Animals (CONCEA).

### *In vivo* model of systemic candidiasis by *Candida krusei*

Inbred female *Balb/c* mice, 6–7 weeks old, were used to evaluate the *in vivo* antifungal activity. A systemic candidiasis model was established according to previously described protocols [28, 29, 38, 39]. A 100 μL cell suspension of *C*. *krusei* ATCC 6258 (1×10^6^ cells) was injected via the lateral tail vein 3 h before the start of antifungal treatment. The infected mice were separated into three groups (n = 5): LMM11 (treated with LMM11 at 5 mg/kg), FLC (treated with fluconazole at 5 mg/kg) and control (treated with diluent, i.e. PBS buffer, DMSO and Pluronic^®^ F-127). All groups were treated twice a day for 5 days by intraperitoneal injection. The mice were euthanized after 5 days and the kidneys were aseptically removed for the determination of fungal burden and histopathological evaluation. The fungal burden analysis of the kidneys was conducted by plating serial dilutions of organ homogenates onto SDA and normalizing the CFU by the weight of the tissue sample (g).

The kidneys for histopathological analysis were immediately fixed in paraformaldehyde 4% for 24 h. The samples were preserved in 70% ethanol, then embedded in paraffin. The kidney was sectioned longitudinally (5 μm sections) and stained using hematoxylin-eosin (HE) and Grocott-Gomori (GG). The presence of fungi and inflammatory cells were analyzed in 20 fields in least in three histological sections. The tissues were observed and photographed using a binocular light microscope (Motic BA310—Moticam 5 camera) at 200x and 600x magnification.

### Statistical analysis

Results were compared using one-way analysis of variance (ANOVA) by applying the Bonferroni multiple-comparisons test and Student’s t-test. The data were analyzed using Prism 6.0 software (GraphPad, San Diego, CA, USA). Values of *p* ≤ 0.05 were considered statistically significant.

## Results

### Antifungal activity of LMM1 against *C*. *krusei*

LMM11 showed inhibitory activity with concentrations ranging from 32 to 64 μg/mL (Table 1). The majority of clinical isolates and the reference strain (89.5%, 17/19) were susceptible to VRC, with the exception of two isolates that were considered resistant (10.5%, 2/19). The MIC for FLC ranged from 4 to 64 μg/mL. *C*. *krusei* is assumed to be intrinsically resistant to FLC and these MICs should not be interpreted using the cut-off levels for susceptible, dose-dependent susceptible and resistant strains. However, LMM11 showed a synergistic effect when combined with fluconazole for both the standard strain and one clinical isolate, with FIC values of 0.75 (Table 2). This result suggests that the combined action of LMM 11 and FLC could be an alternative for the treatment of resistant species.

**Table 1:** Antifungal susceptibility of *C*. *krusei* isolates (n = 18) and reference strain *C*. *krusei* ATCC 6258 to conventional antifungal agents and LMM11.

Antifungal agent	MIC (μg/mL)				N (%)	
	Range	MIC_50_	MIC_90_	S	SDD	R
Fluconazole ^a^	4–64	32	32	-	-	-
Voriconazole	0.25–2	0.5	0.5	17 (89.5%)	0	2 (10.5%)
LMM11	32–64	32	64	-	-	-

Abbreviations; MIC: minimal inhibitory concentration; R: resistant; S: susceptible; SDD: susceptible-dose dependent. MIC_50_ and MIC_90_ were defined as the antifungal concentration capable of inhibiting the growth of the isolates by 50% and 90%, respectively.

^a^ isolates of *C*. *krusei* are assumed to be intrinsically resistant to fluconazole and their MICs should not be interpreted using this scale.

**Table 2:** Combined antifungal effect of LMM11 and fluconazole against *Candida krusei* ATCC 6258.

Drugs	Strains	MIC (μg/mL)		FIC^A^	MIC (μg/mL)		FIC^B^	FIC	IN
		MIC^A^_Comb_	MIC^A^_Alone_		MIC^B^_Comb_	MIC^B^_Alone_			
FLU+LMM11	ATCC *C*. *krusei* 6258	8	32	0.25	32	64	0.5	0.75	S
	Clinical isolate 274	16	32	0.5	8	32	0.25	0.75	S

Abbreviations; MIC: minimal inhibitory concentration; FLC: fluconazole; MIC^A^_comb_: FLC MIC when used in combination with LMM11; MIC^A^_alone_: FLC MIC when used alone; MIC^B^_comb_: MIC of LMM11 when used in combination with FLC; MIC^B^_alone_: LMM11 MIC when used alone; FIC: fractional inhibitory concentration; IN: interpretation; S: strongly synergistic effect; FIC^A^: MIC^A^ combined/MIC^A^ alone; FIC^B^: MIC^B^ combined/MIC^B^ alone; FIC: FIC^A^ + FIC^B^.

**Fig 1:**
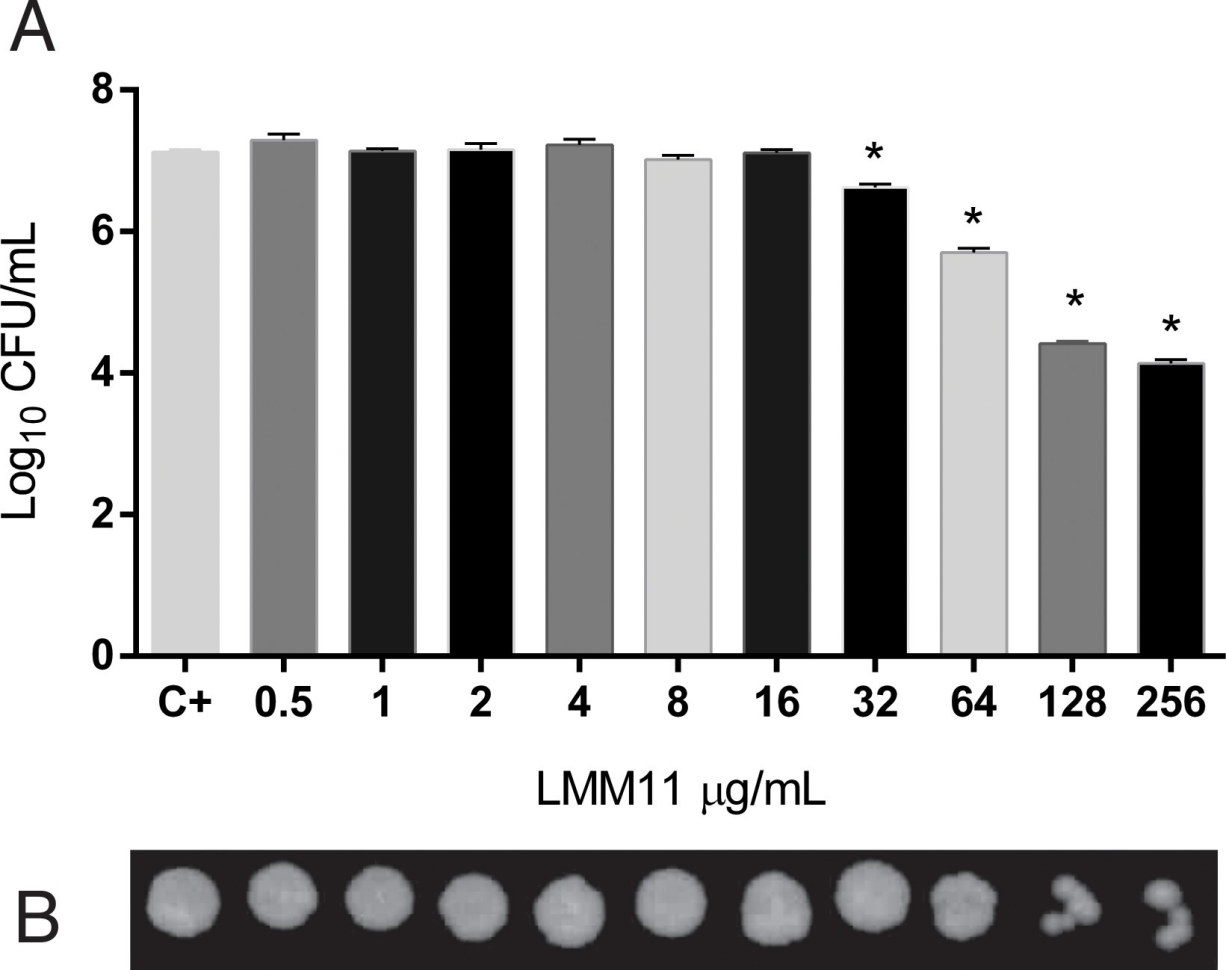
Quantitative and qualitative analysis of the effect of LMM11 on *C*. *krusei* ATCC 6258. (A) Logarithmic reduction of colony forming units (CFU) and (B) minimum fungicidal concentration (MFC) after exposure to increasing LMM11 concentrations for 24 hours. C+: Positive control (inoculum without LMM11).

The results on the MFC demonstrated a dose-dependent effect of LMM11 against *C*. *krusei* (Fig 1B). A significant reduction in CFU was observed at 32 μg/mL (*p*<0.05) after 24 h of incubation with LMM11 in relation to the control (Fig 1A). At the two highest concentrations tested, the reduction was ~3 log_10_.

### Time-kill curve

**Fig 2:**
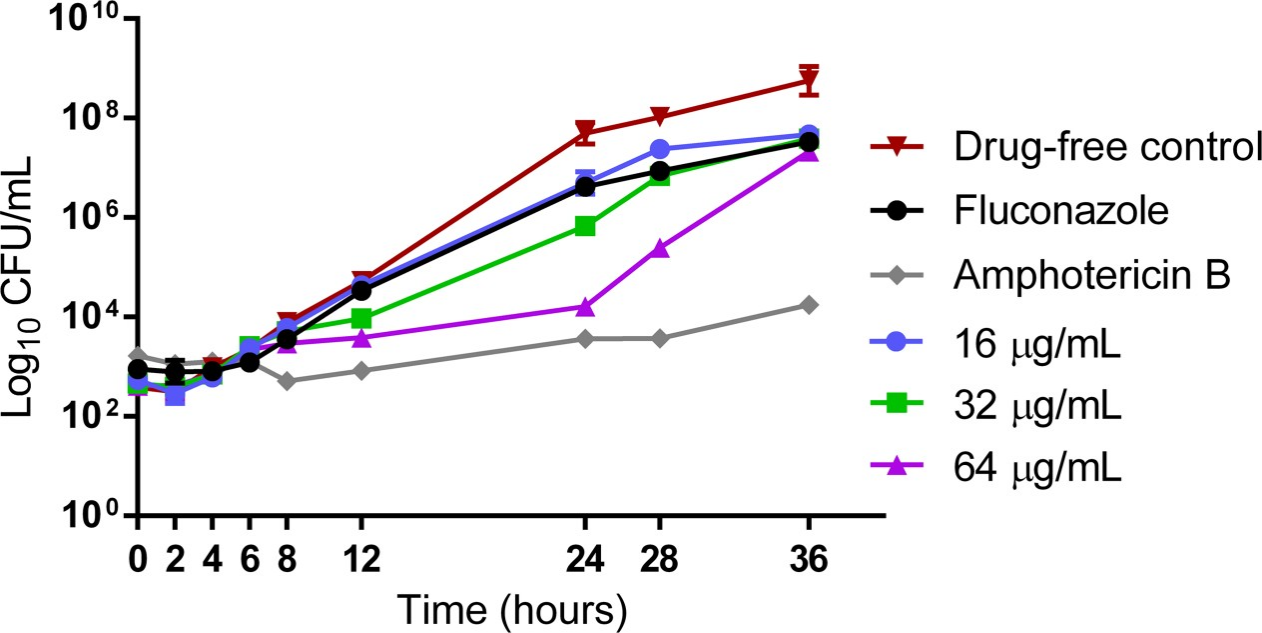
LMM11 time-kill curve against *C*. *krusei* ATCC 6258. Standardized yeast cell suspensions were exposed to 16 μg/mL, 32 μg/mL and 64 μg/mL LMM11. Fluconazole (16 μg/mL) and amphotericin B (0.25 μg/mL) were used as conventional drug controls. An additional control was incubated in the absence of LMM11 (drug-free control). At determined time intervals, samples were serially diluted and plated on SDA for the determination of CFU. Each data point represents the mean ± standard deviation (error bars).

The killing activity of LMM11 (16 μg/mL, 32 μg/mL and 64 μg/mL), FLC (16 μg/mL) and AMB (0.25 μg/mL) plotted from log_10_ CFU/mL versus time (36 h) is represented in Fig 2. The inhibitory effect of LMM11 began 8 h after the start of incubation. The best activity of this compound in relation to the drug-free control was observed at 24 h. At this point of the time-kill curve, the activity profile of LMM11 was similar to that obtained with the conventional drug AmB. The concentration of 64 μg/mL resulted in a >99.9% reduction (~4 log_10_) in the number of viable cells. FLC showed little activity against *C*. *krusei*, highlighting the resistance of this species to this antifungal agent. LMM11 appeared to exhibit fungicidal activity against *C*. *krusei*.

### *C*. *krusei* cell viability after LMM11 exposure

**Fig 3:**
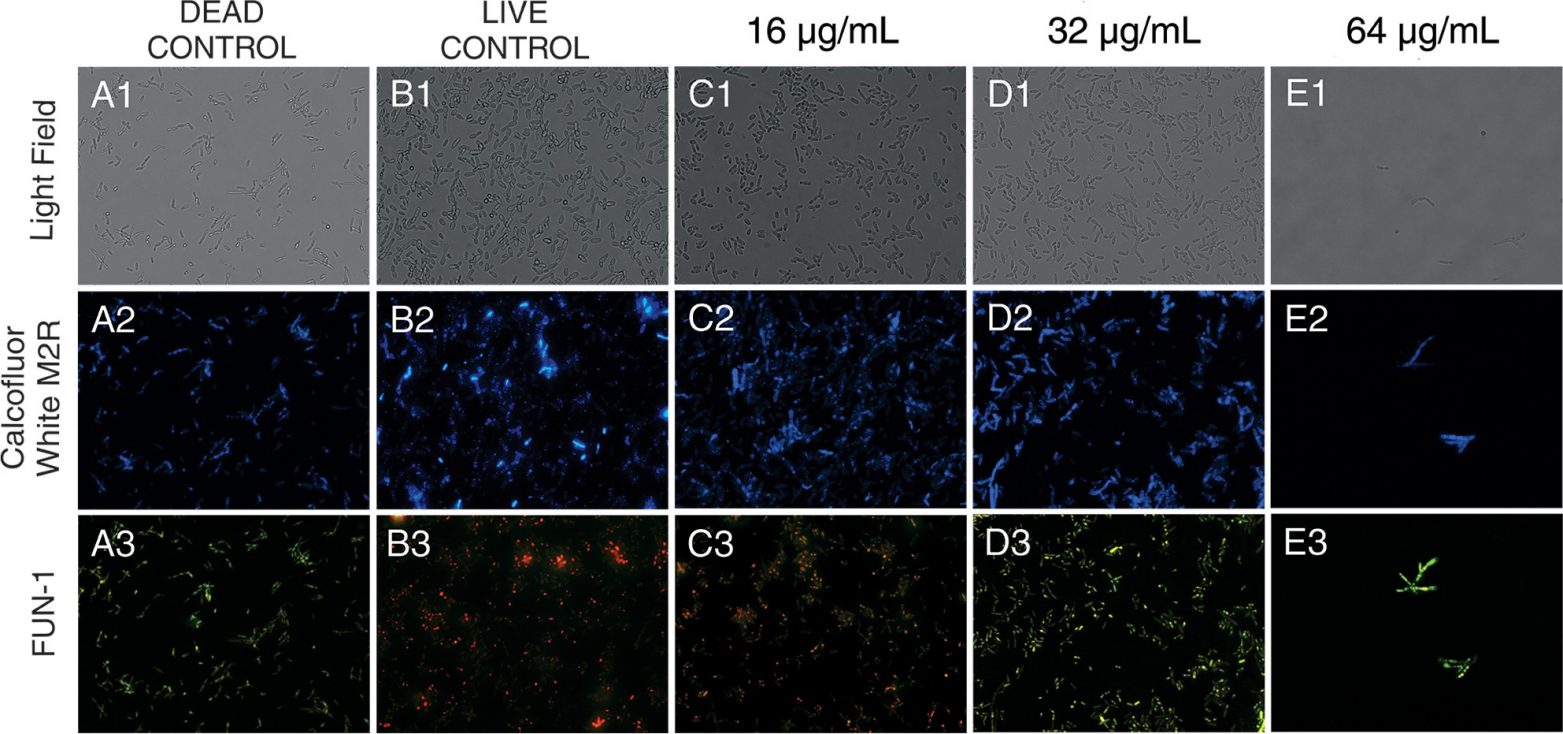
Cell viability assay of *C*. *krusei* ATCC 6258 after treatment (24 h) with LMM11 at 16 μg/mL, 32 μg/mL and 64 μg/mL. In metabolically active cells, the cytoplasm presents diffusely distributed green fluorescence and contains cylindrical red fluorescent structures in vacuoles. Dead cells or cells with little or no metabolic activity present bright yellow-green fluorescence with no discernable red structures. Dead control (A1-A3): yeast treated with 70% alcohol for 15 minutes. Live control (B1-B3): untreated yeast. Brightfield images (A1, B1, C1, D1 and E1). Yeast were also stained with Calcofluor (A2, B2, C2, D2 and E2). Yeast treated with 16 μg/mL (C3) maintained a pattern of cellular metabolism similar to the live control (B3). However, 32 μg/mL (D3) and 64 μg/mL (E3) LMM11 caused a marked decrease in cell number and cell viability. The samples were observed at 400x magnification. Analysis was performed on at least 20 fields. The assays were performed the using LIVE/DEAD yeast viability kit (L7009).

Yeast cells treated with LMM11 at a concentration of 16 μg/mL (Fig 3C3) and the live control (Fig 3B3) were marked with a diffusely distributed green fluorescence and presence of cylindrical red-fluorescent structures in their vacuoles indicating that these cells were metabolically active. However, the samples treated with 32μg/mL (Fig 3D3), 64μg/mL (Fig 3E3) and dead control (Fig 3A3) showed fluoresce bright yellow-green, with no discernable red structures, indicating that those have died or had low or no metabolic activity. The fluorescence of intravacuolar structures which indicates metabolically active cells requires both plasma membrane integrity and metabolic capability. Complementing the above observations, it was verified cell population reduction according to the increase of the LMM11 concentration as compared to control.

### Scanning electron microscopy

**Fig 4:**
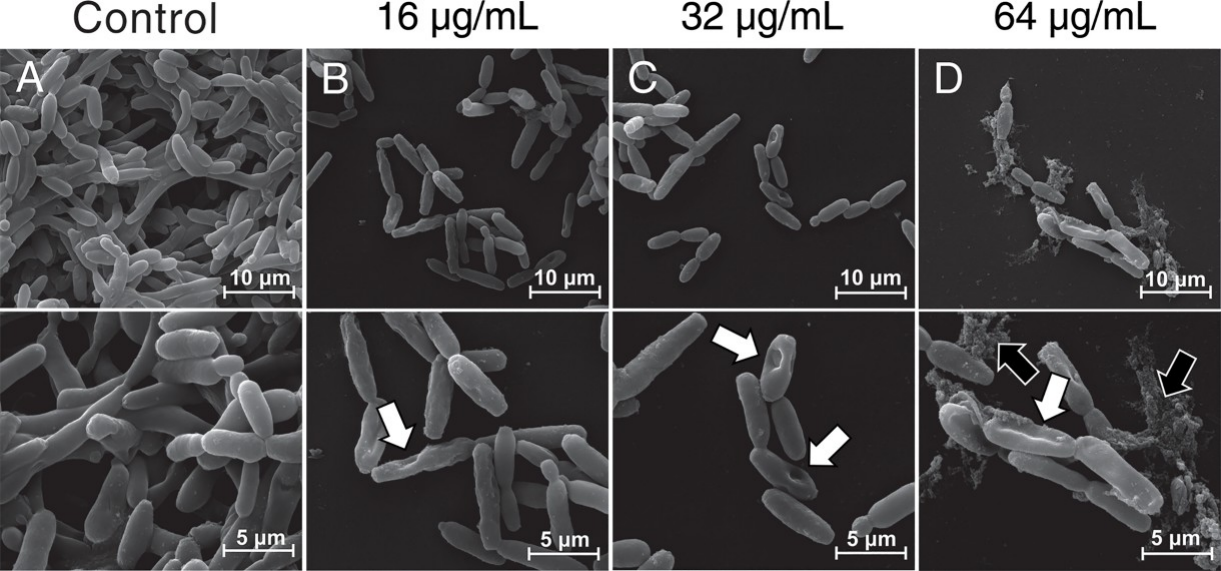
Scanning electron microscopy of *C*. *krusei* ATCC 6258 after exposure to LMM11. Yeasts were incubated with LMM11 for 24 h at 35°C. Control (A) not exposed; (B) 16 μg/mL; (C) 32μg/mL; (D) 64 μg/mL. White arrows indicate depressions on the cell surface and black arrows indicate extravasation of cellular contents. The samples were observed at 6000x and 12000x magnification. The analysis was performed on at least 20 fields.

SEM was used to analyze morphological alterations in *C*. *krusei* treated with LMM11 at 16 μg/mL, 32 μg/mL and 64 μg/mL (Fig 4). The untreated control showed normal yeast morphology (Fig 4A). Loss of cell integrity with extravasation and retractions in the cell surface were observed at all tested concentrations (Fig 4B–4D). Morphological alterations were dose-dependent. Although this assay is not a quantitative analysis, it was possible to observe a reduction in the cell population with an increase in the LMM11 concentration compared to the control.

### Transmission electron microscopy

**Fig 5:**
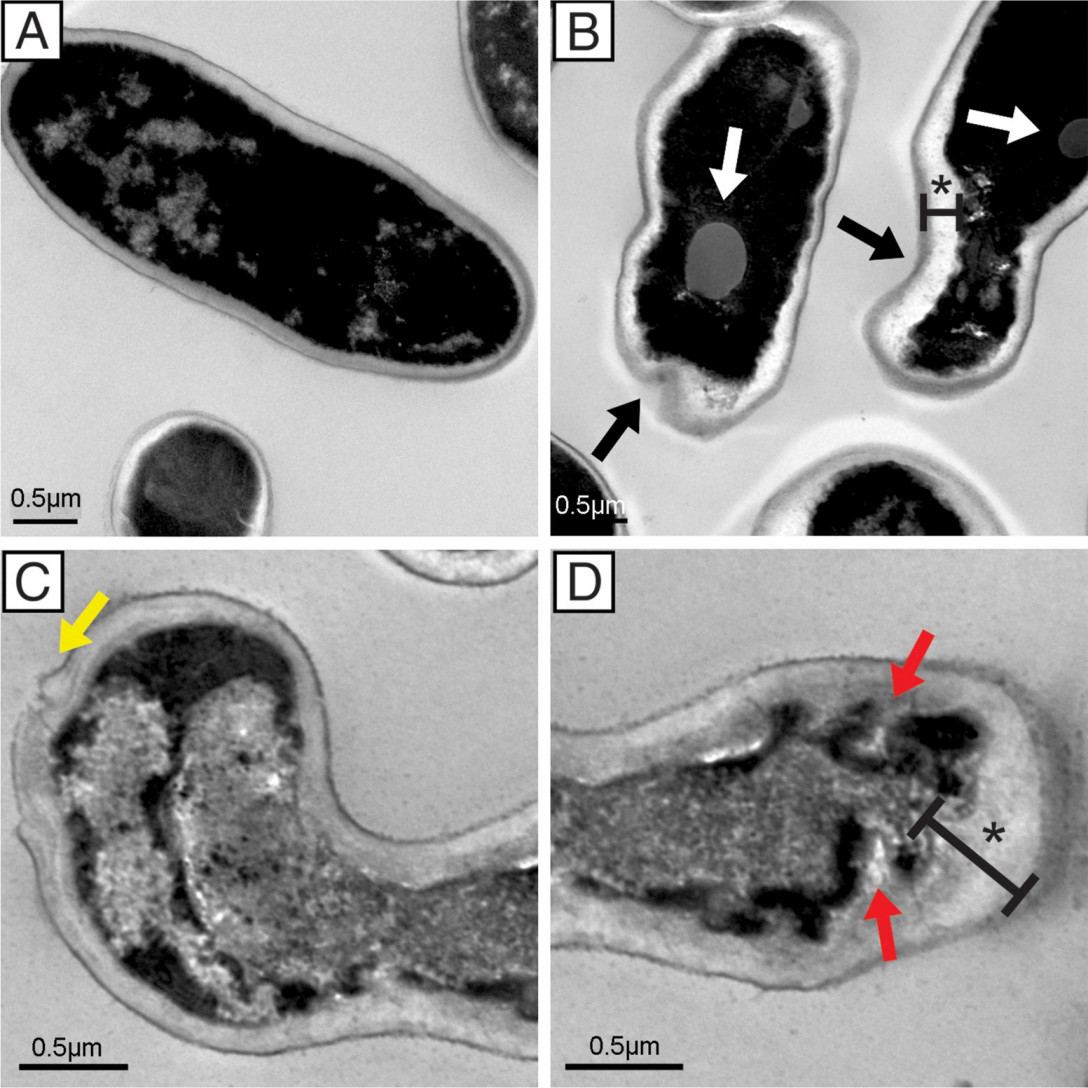
Representative transmission electron microscopy micrographs of *C*. *krusei* ATCC 6258. Yeast were incubated with 32 μg/mL LMM11 for 24 h at 35°C. Untreated cells had a normal appearance (A). Treated cells (B-D) presented ultrastructural alterations such as invaginated cell membrane (red arrows), irregular cell wall surfaces (black arrows), cytoplasmic retractions (asterisk), lipid vacuoles (white arrows) and cellular extravasation (yellow arrows), which were not observed in the control. The samples were observed at 25000x magnification. The analysis was performed on at least 20 fields. Bars = 0.5 μm.

The TEM analysis corroborated the SEM results. Surface changes were also observed in the ultrastructural analysis. *C*. *krusei* cells treated with LMM11 presented significant changes that led to the total destruction of fungal cells (Fig 5B–5D). The most frequently observed ultrastructural alterations were irregular cell wall surfaces, loss of cell wall integrity with apparent extravasation of cellular components (Fig 5C), cytoplasmic membrane with the presence of invaginations and cytoplasmic retraction resulting in an increased gap between the cell wall and the plasma membrane (Fig 5D). Loss of electron density and the presence of lipid vacuoles were also evident (Fig 5B). The untreated yeast (control) presented a continuous cytoplasmic membrane and cell wall integrates. (Fig 5A).

### LMM11 as a promising alternative for systemic candidiasis treatment

**Fig 6:**
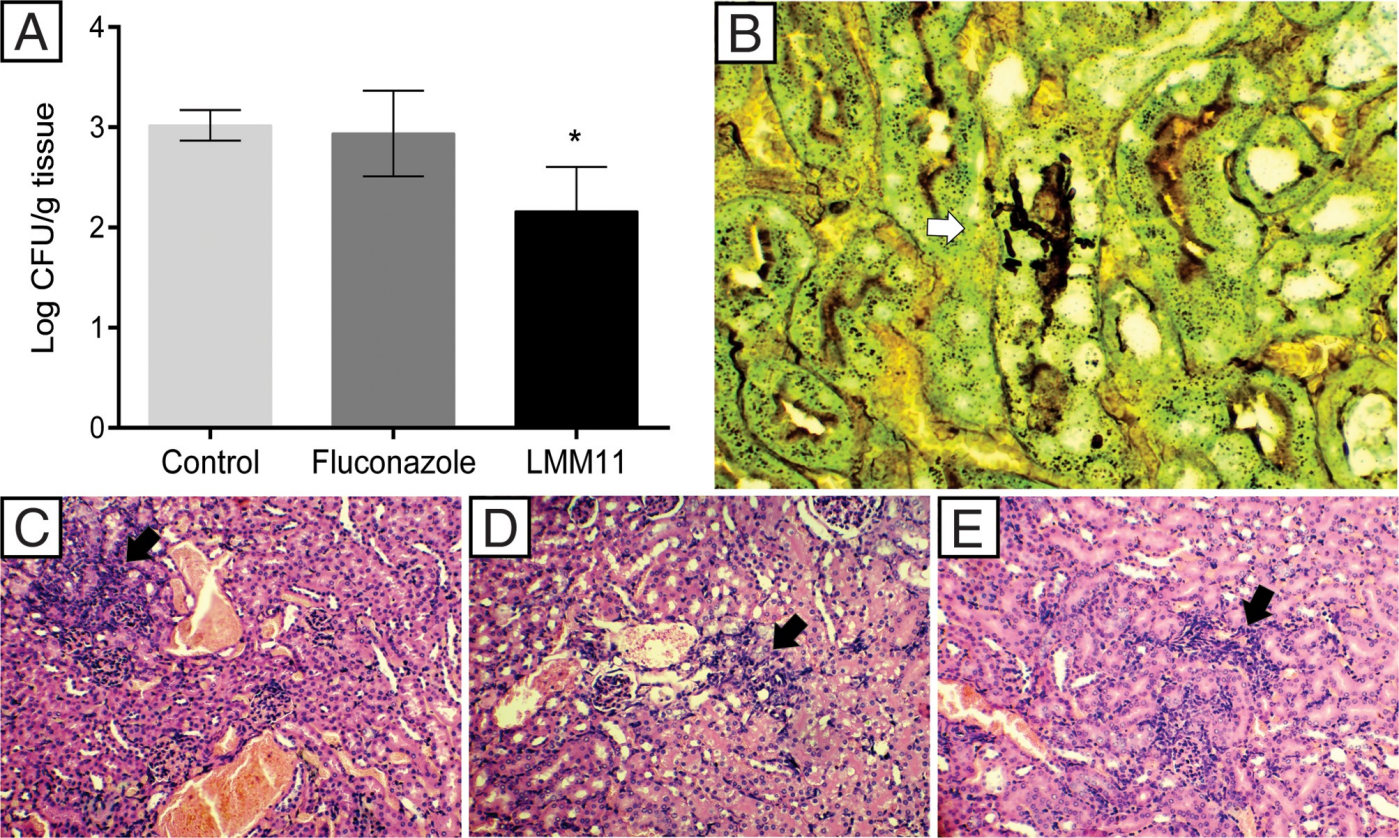
The *in vivo* efficacy of LMM11 in systemic *C*. *krusei* candidiasis. Mice were infected with *C*. *krusei* ATCC 6258 (1x10^6^ cells) and separated into three groups (n = 5): control (treated with PBS and the diluent), FLC (treated with fluconazole at 5 mg/kg) and LMM11 (treated with LMM11 at 5 mg/kg). Drugs were given to the mice twice a day for 5 days via intraperitoneal injection. (A) Colony forming units (log_10_ CFU) per gram of kidney. LMM11 significantly reduced the renal fungal burden in relation to the control and FLC (*p<0.05). The bars indicate the standard deviation. (B) Kidney histological sections after Gomori-Grocott staining to indicate the presence of yeast (white arrow) only in the control group. (C-E) Histological sections stained with hematoxylin and eosin showing inflammatory infiltrate (black arrow) in the control (C), FLC (D) and LMM11 (E). Representative kidney histopathological sections from 5 mice per group.

In order to assess the *in vivo* efficacy of LMM11 to treat systemic candidiasis by *C*. *krusei*, Balb/c mice were treated twice daily for five days with LMM11 (5 mg/kg). LMM11 was efficient in reducing the kidney fungal burden (0.85 log_10_ CFU/g) when compared to the control group (p <0.05; Fig 6A). The FLC and control groups presented similar behavior and no reduction in fungal burden. Gomori-Grocott staining revealed the presence of fungal yeast structures in the renal tissue in the control group (Fig 6B). However, yeasts were not visualized in the groups that were treated with either FLC or LMM11. Kidney histology by hematoxylin-eosin staining revealed inflammatory alterations in all groups, which were more intense in the control group (Fig 6C) than in the groups treated with FLC (Fig 6D) or LMM11 (Fig 6E).

**Fig 7:**
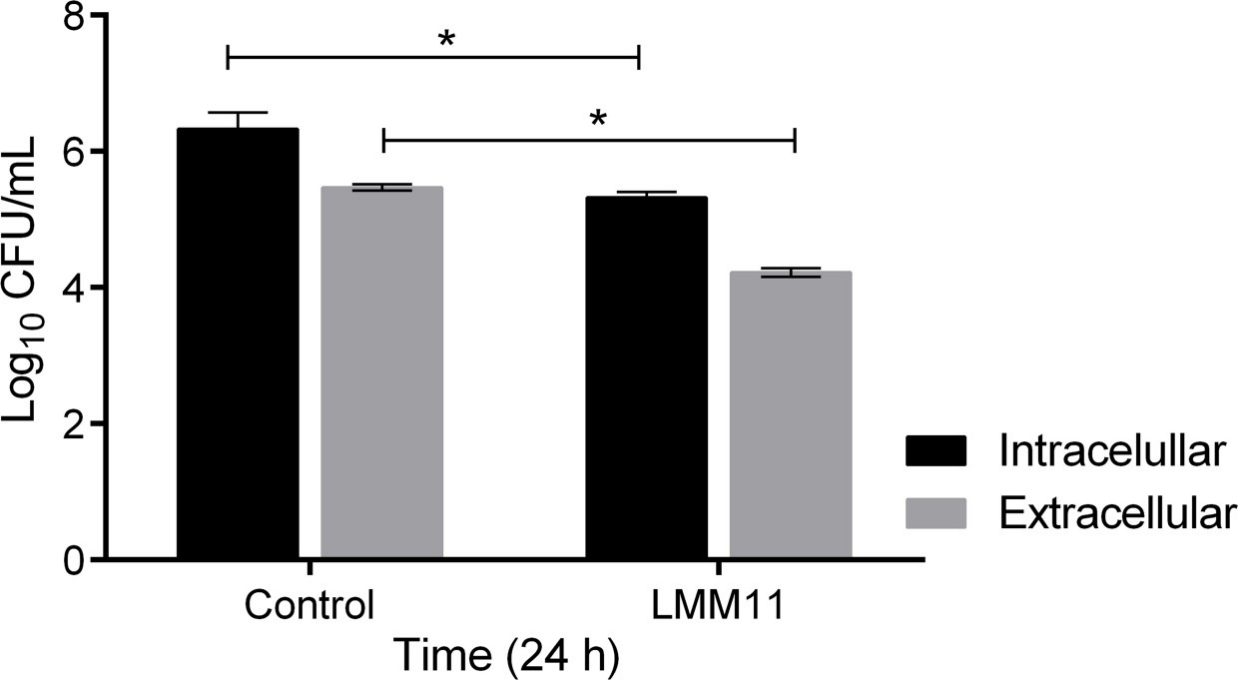
Antifungal activity of LMM11 against intracellular and extracellular *C*. *krusei* after phagocytosis by J774-A1 macrophages. After 24 hours, supernatants were collected for the CFU determination of extracellular cells. Alternatively, macrophages were lysed for the CFU determination of intracellular cells. (LMM11) Macrophages with internalized *C*. *krusei* cells and incubated with LMM11 at concentration 32 μg/mL. (Control) Macrophages with internalized *C*. *krusei* cells and incubated with RPMI-1640 only (untreated). *p<0.05, statistically significant values comparing extracellular or intracellular treated versus extracellular or intracellular untreated. The bars indicate the standard deviation.

*C*. *krusei* is able to survive and replicate within phagocytic cells; this characteristic can determine the susceptibility of the host to infection. *In vivo* treatment efficacy was enhanced by the intracellular effect of LMM11. After 24 hours, the CFU analysis revealed a significant decrease in *C*. *krusei* replication inside and outside macrophages in relation to the control (p <0.05; Fig 7). Thus, these results collaborate with the *in vivo* treatment efficacy, as a significant reduction in fungal burden was observed.

## Discussion

*C*. *krusei* is a notorious pathogen that has been recognized as a potentially multidrug-resistant (MDR) fungus due to its intrinsic resistance to FLC and reduced susceptibility to other azoles and amphotericin B [16]. Therefore, in view of the few antifungal agents available, the search for more specific alternative therapies is becoming increasingly necessary, but drug development by conventional methods requires 12 to 15 years until commercialization and costs can reach more than one billion dollars [40]. Rational drug design involves the use of computational tools in drug discovery as a cost-effective alternative to traditional experimental protocols [41]. Several research groups have shown the excellent antifungal potential of compounds selected by virtual screening of small molecule libraries against specific targets [26, 39, 42–47].

Salci *et al*. [46] showed fungicidal activity against *C*. *albicans*, *C*. *parapsilosis*, *C*. *tropicalis* and *C*. *krusei* with MOL3 (a KRE2 inhibitor) selected by virtual screening. In another study, a yeast-to-hypha transition inhibitor for *C*. *albicans* revealed good *in vitro* activity against *C*. *krusei*, although the *in vivo* activity was not evaluated [39]. In the present study, the activity of LMM11 *in vitro* and *in vivo* against *C*. *krusei* was determined. Although LMM11 was selected based on the target TRR1 from *C*. *albicans*, our results demonstrate antifungal activity against *C*. *krusei*. Previous studies in our laboratory have shown that this compound has a broad spectrum of action, especially against fungal pathogens such as other *Candida* species, *Paracoccidioides* spp. and *Cryptococcus* spp., with low toxicity [27–29].

Interestingly, LMM11 showed stable antifungal activity between 12–24 h with efficient reduction of yeast viability (>99.9%). The time-kill curve results show that LMM11 antifungal activity was compatible with AmB. Thus, the LMM11 profile found at 24 h against *C*. *krusei* seems to be fungicidal. These results corroborate the *in vitro* studies, especially the structural microscopy assessments. Although still a hit compound, this profile is quite promising for the development of new therapeutic options. The ultrastructural analysis of yeast exposed to LMM11 revealed important morphological alterations that indicated cellular destruction, loss of cellular contours and growth inhibition. The formation of depressions on the cell surface (retractions) and cell shrinkage were also observed in the images and were more marked with increasing drug concentrations. Similar morphological changes to *C*. *krusei* were observed in a study using silver nanocompounds. These irregularities were attributed to the ability of the compound to cause cell membrane damage [48]. Another study that evaluated the antifungal effect of the tripeptide FAR (Phe-Ala-Arg) against *C*. *krusei* showed similar irregularities on the fungal cell surface due to peptide accumulation in the membrane, causing an increase in permeability and loss of barrier function, which led to cell death [49].

The alterations observed in yeast cells treated with LMM11 might be due to cell wall damage as well as in the cytoplasmic membrane, resulting in the loss of intracellular compounds and complete cell disruption. These results are in agreement with those found in the LIVE/DEAD assay and by TEM, which also revealed changes in the wall and cell membrane. Furthermore, these findings suggest that LMM11 may damage or disrupt the cellular structure of yeast, resulting in fewer cells that retain metabolic activity. However, the details of the mechanism of action of this compound are still not understood. Further studies are needed to fully elucidate the antifungal pathways of LMM11.

Treating *Candida* infections with monotherapy is becoming more difficult due to increased antifungal resistance [16, 50, 51]. Combination therapy may be a therapeutic solution against resistant species. Many studies have focused on synergistic effects as an alternative to the antifungal agents developed against *Candida* spp. [52–57]. However, there is a range of protocols with different interpretations [37, 58–62]. In this study, we evaluated the synergistic effect of LMM11 with FLC against *C*. *krusei* and interpreted the results according to the Mor *et al*. [37]. Our results show that LMM11 can be successfully combined with FLC against *C*. *krusei*, extending its spectrum of action and providing an alternative approach to overcoming antifungal drug resistance. Although the mechanism of this synergistic activity is not understood, it could be inferred that the weakening fungal membrane structures by FLC may facilitate LMM11 penetration, thus augmenting antifungal activity, since this compound acts by inhibiting thioredoxin reductase (TRR1), which is located in the cytoplasm and plays a critical role in maintaining the redox state of the cell.

In addition to intrinsic resistance to FLC, another aspect of *C*. *krusei* infection is the ability of the fungus to survive in and exploit the intracellular environment of macrophages for replication, which may influence dissemination through the organism [63]. LMM11 was effective against phagocytosed *C*. *krusei* in our model. The capacity to exert antifungal activity on phagocytosed yeast cells is particularly important as these results suggest that LMM11 could be used to overcome the initial failure of systemic *C*. *krusei* candidiasis treatment.

The murine model of systemic *Candida* infection has been used extensively to study host defense and antifungal drug efficacy [39, 46, 64]. The kidney is one of the main target organs of disseminated *Candida* infection in mice [64]. Therefore, the kidney fungal burden was evaluated in this study. In our model employing systemic *C*. *krusei* infection in immunocompetent mice, FLC treatment was not efficient, diverging from the results found by Graybill *et al*. [65]. However, our data corroborate those found in patients, in whom *C*. *krusei* is less responsive to FLC [66]. Although the inflammatory alterations remained practically the same in all treated groups, LMM11 demonstrated efficacy in systemic *C*. *krusei* infection treatment with a significant reduction in the kidney fungal burden showing better results than those obtained with the conventional drug. Taken together, our results indicate that LMM11 has excellent potential for the development of an antifungal agent for the treatment of species resistant to conventional antifungal drugs.
